# Supplementary material for: Improving forest ecosystem functions by optimizing tree species spatial arrangement
Source: Nat Commun. 2025 Jul 9;16:6286. doi: 10.1038/s41467-025-61389-7 (PMC12241584; doi:10.1038/s41467-025-61389-7)
Supplement: Supplementary file 1 — Supplementary Information [file 41467_2025_61389_MOESM1_ESM.pdf]

Supplementary Information

Supplementary Table S1

Species	Myc type	Biomass (10^3kg)	SLA (mm2/mg)	LDMC (mg/g)	CN	C (%)	N (%)
Castanea henryi	ECM	0.010700	16.709402	443.6912	25.09235	47.72935	1.913439
Castanopsis sclerophylla	ECM	0.002910	10.541827	455.5130	31.75763	48.51781	1.537868
Choerospondias axillaris	AM	0.026800	19.698164	372.1320	25.66076	48.31840	1.997127
Cyclobalanopsis glauca	ECM	0.002500	9.288378	521.7610	33.78928	49.33557	1.506321
Koelreuteria bipinnata	AM	0.001530	15.649323	422.8349	31.10405	47.88557	1.761470
Liquidambar formosana	AM	0.005250	17.441354	350.2780	30.52753	46.86200	1.647137
Lithocarpus glaber	ECM	0.006110	8.843517	503.2277	36.96489	49.69844	1.263288
Nyssa sinensis	AM	0.010400	20.897022	342.0438	25.15585	46.82723	1.885227
Quercus fabri	ECM	0.000589	17.028665	511.3304	26.11837	48.24561	1.963446
Quercus serrata	ECM	0.001390	20.073847	426.7143	22.96407	47.63995	2.024518
Sapindus mukorossi	AM	0.003890	22.563375	334.0061	20.50625	46.08773	2.293581
Sapium sebiferum	AM	0.002880	19.199424	329.7754	21.78281	46.39627	2.126776

Supplementary Note S1: Simulated plot design

Number of species per plot

We constructed plots featuring mixtures of 2, 4, and 8 different species to create evenly distributed groups of species within 16 by 16 tree plots.

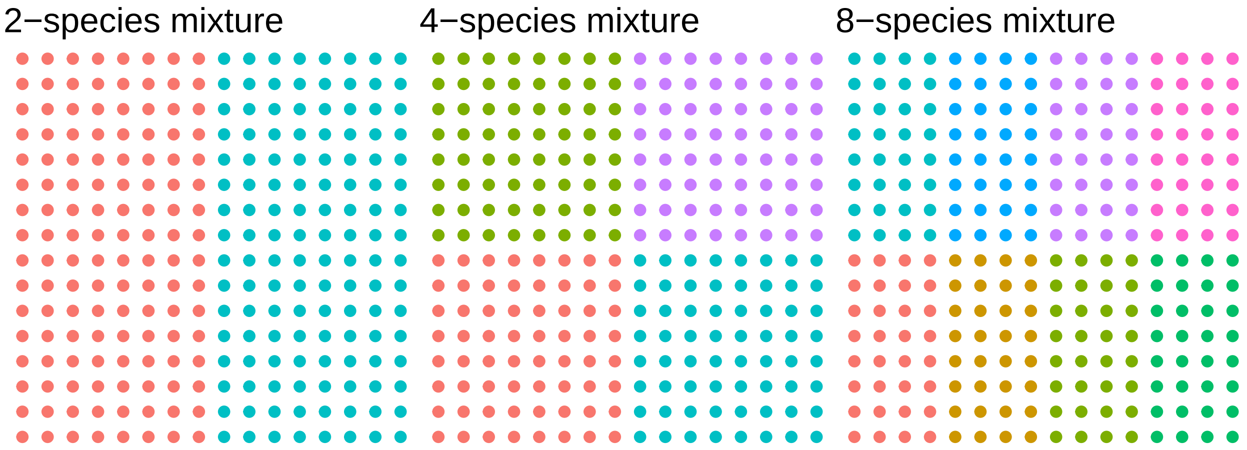

Supplementary Figure S1: Block spatial distribution of the tree species (colored dots), in 2-, 4- and 8-species mixtures.

Permutation gradient

8-species mixtures

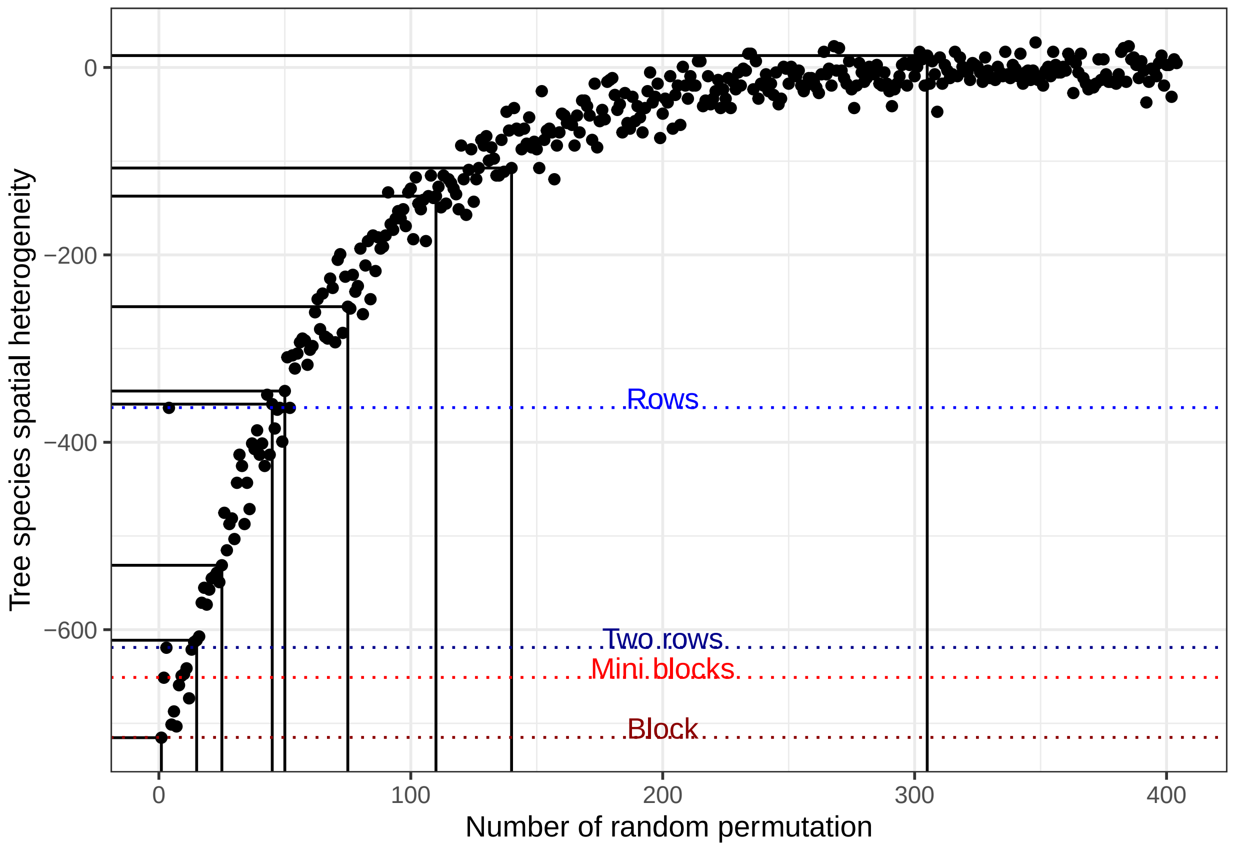

Supplementary Figure S2: tree species spatial heterogeneity as a function of random permutation of the tree species in 8-species mixture. Black lines highlight selected permutations and colored lines the levels of heterogeneity for block, mini-block, rows, and two rows designs.

4-species mixtures

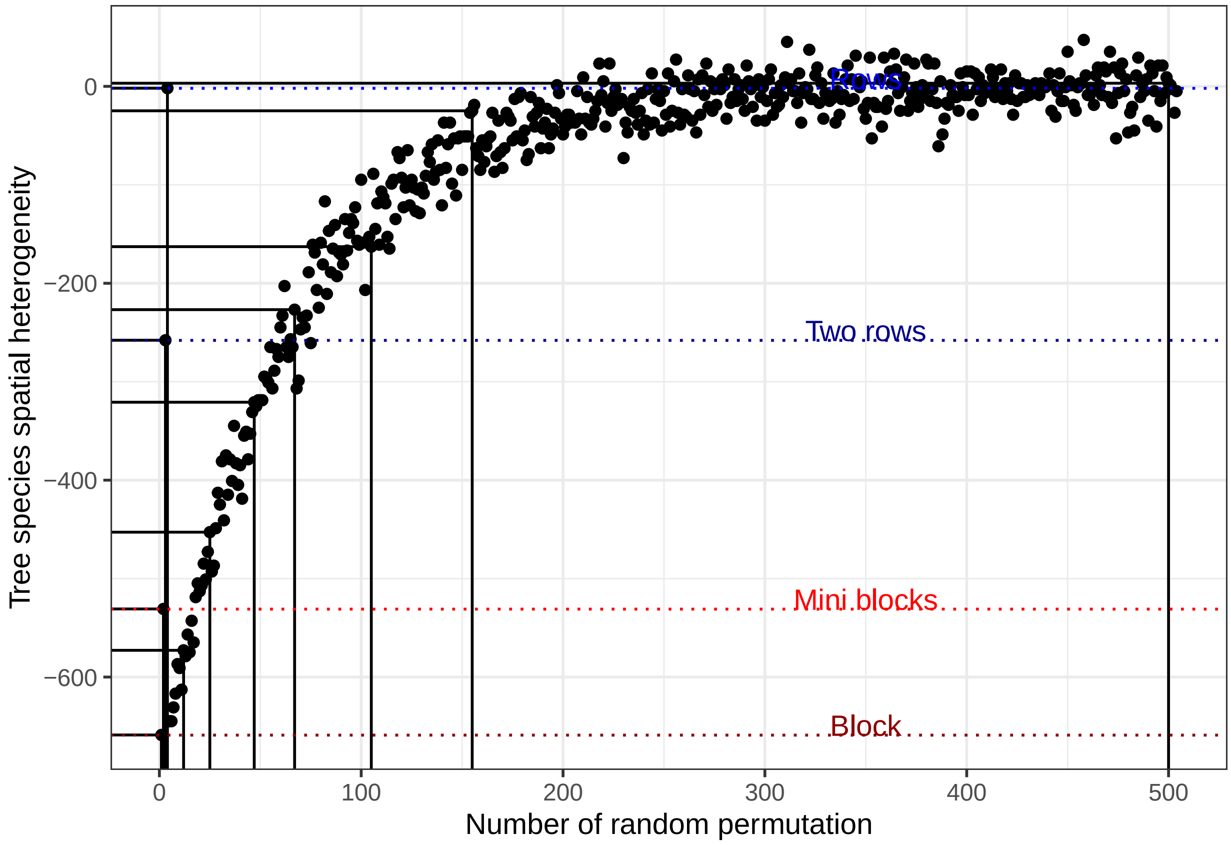

Supplementary Figure S3: tree species spatial heterogeneity as a function of random permutation of the tree species in 4-species mixture. Black lines highlight selected permutations and colored lines the levels of heterogeneity for block, mini-block, rows, and two rows designs.

2-species mixtures

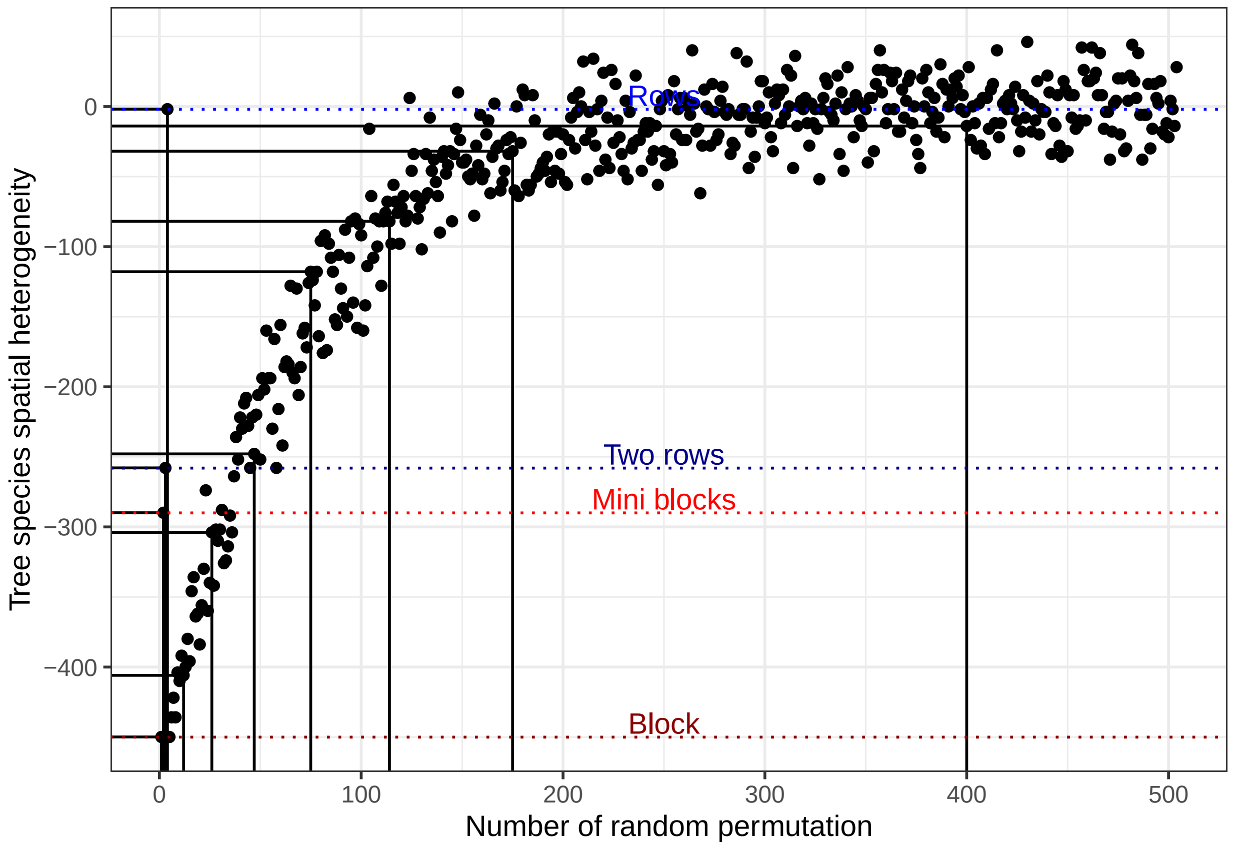

Supplementary Figure S4: tree species spatial heterogeneity as a function of random permutation of the tree species in 2-species mixture. Black lines highlight selected permutations and colored lines the levels of heterogeneity for block, mini-block, rows, and two rows designs.

Plot designs

Plot spatial designs selected for the study. Colored dots represent the species distributed on the 16 by 16 tree forests

8-species mixtures

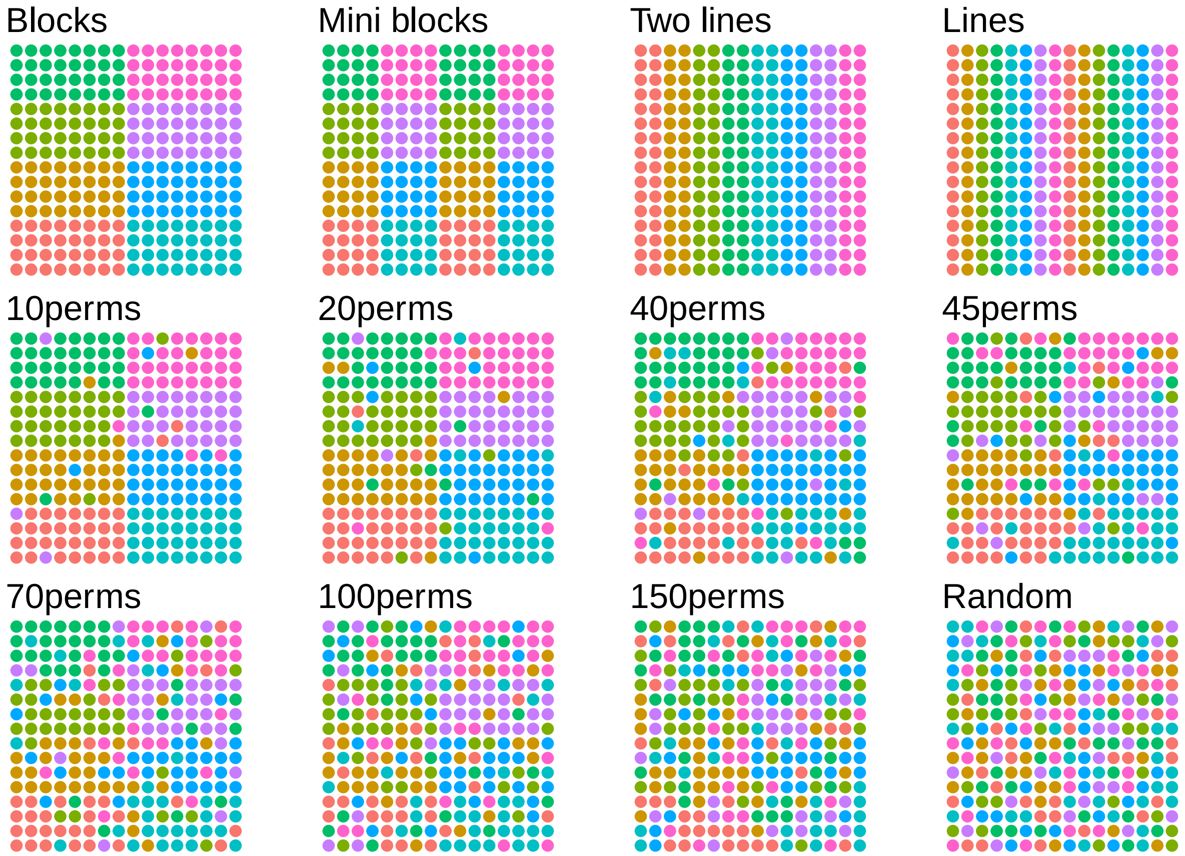

Supplementary Figure S5: selected 8-species mixtures plot design. Each color reprerent a different species.

4-species mixtures

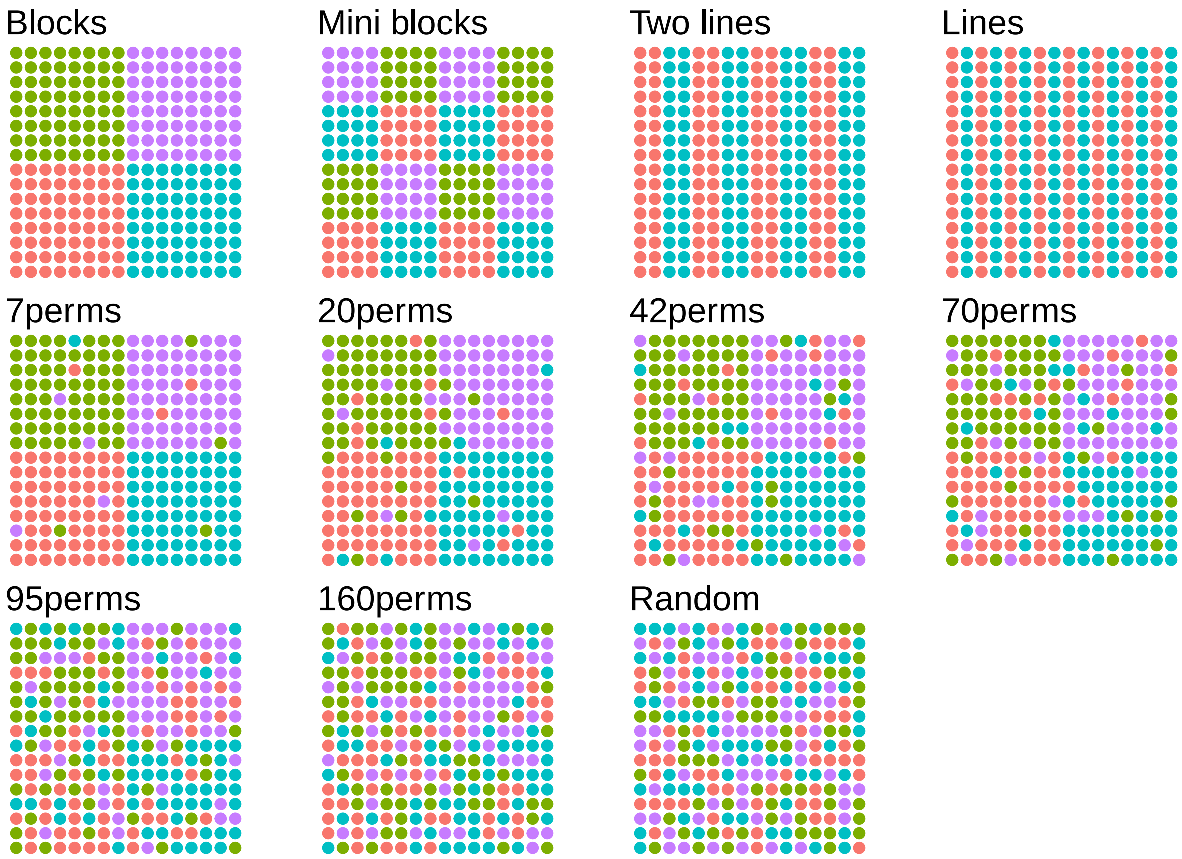

Supplementary Figure S6: selected 4-species mixtures plot design. Each color reprerent a different species.

2-species mixtures

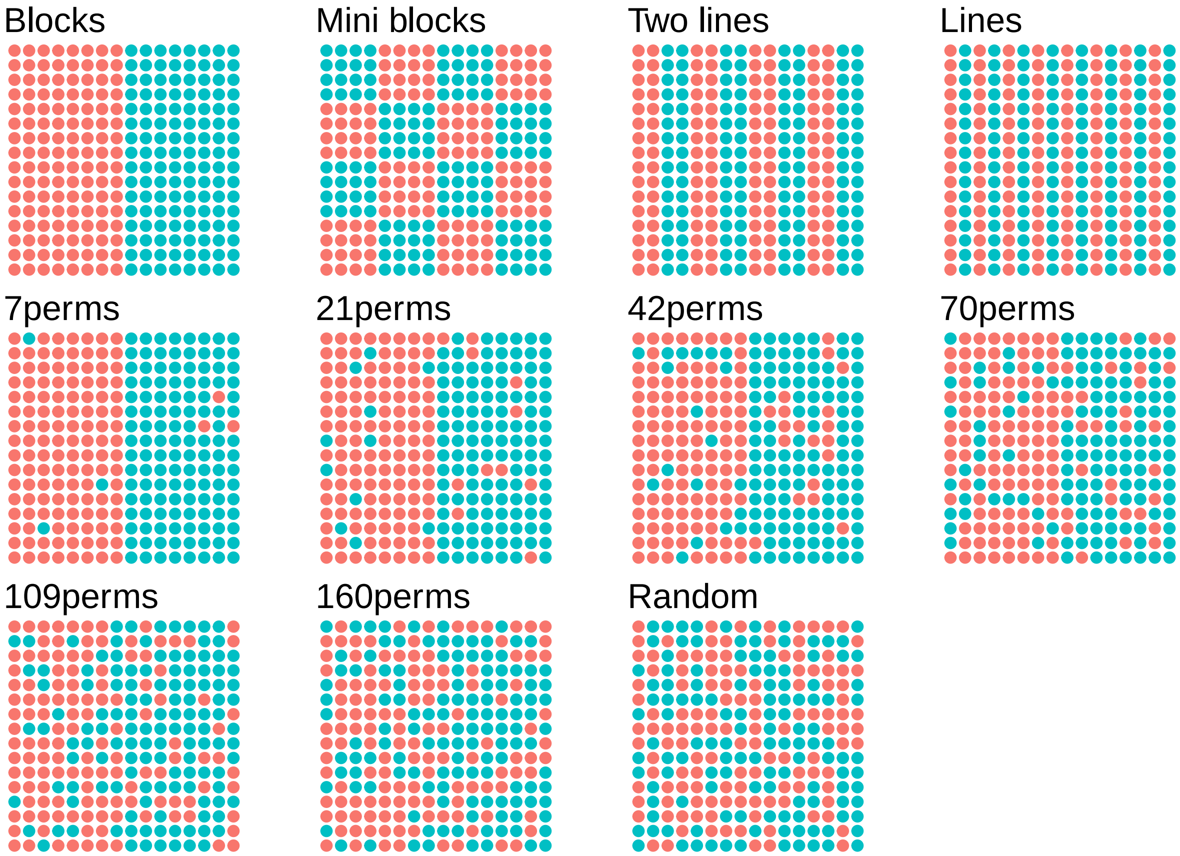

Supplementary Figure S7: selected 2-species mixtures plot design. Each color reprerent a different species.

Supplementary Note S2: litterfall and decomposition statistical models

Litterfall distribution models

Input variables .

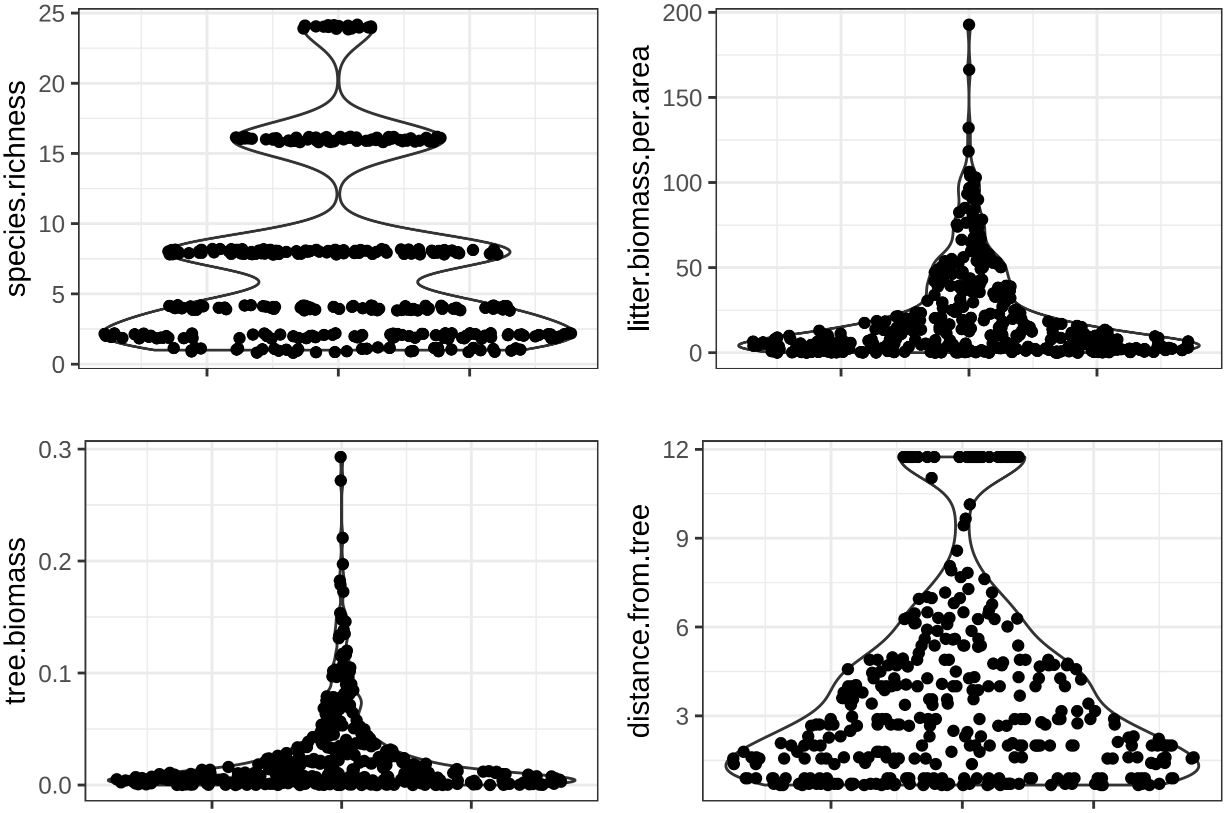

Supplementary Figure S8: explanatory variables distribution.

Model structure

```
stan_code = '
data{
  int n; // observations per species
  int m; // species number
  matrix[m,n] litter; // Species specific litter
  matrix[m,n] biomass;  // Species specific biomass
  matrix[m,n] dist;  // Species specific distance
  matrix[m,n] biodist;  // Species specific distance
}

parameters{
  vector<lower=0>[m] bio; // Species specific biomass parameter
  vector<lower=0>[m] d; // Species specific distance parameter
  vector<lower=0>[m] db;  // Species specific interraction parameter
  vector<lower=0>[m] sigma;  // Species specific sigma
}

model{
  // Priors
  matrix[m,n] mu;  // Species specific mu
  bio ~ normal(0,10); // Species specific biomass parameter prior
  d ~ normal(0,10);  // Species specific distance parameter
  db ~ normal(0,10);  // Species specific interaction parameter
  sigma ~ normal(0,10); // Species specific sigma

  // Likelihood
  for(i in 1:n){
    for(j in 1:m){
      mu[j,i] = bio[j] * biomass[j,i] + d[j] * dist[j,i] + db[j] * biodist[j,i] ;
      //Species specific litterfall
      litter[j,i] ~ normal(mu[j,i], sigma[j]);
    }
  }
}
'
```

Model fit

Posterior distribution

Biomass parameters .

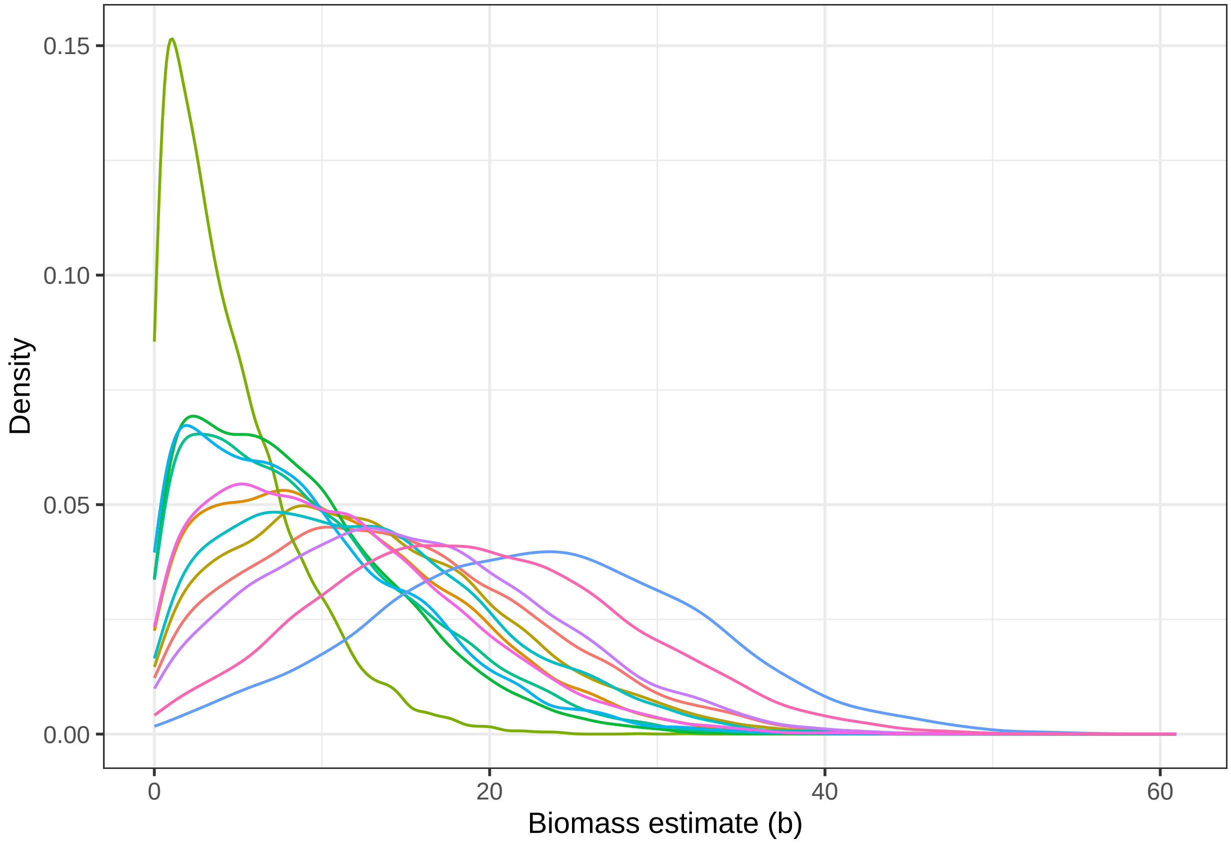

Supplementary Figure S9: posterior distribution of tree species biomass effect on litterfall (each color represent a species).

Average estimate:

```
##    bio[1]    bio[2]    bio[3]    bio[4]    bio[5]    bio[6]    bio[7]    bio[8]
## 14.272563  8.509328  9.203823 12.350001  8.910117 22.992980 15.206351 10.854432
##    bio[9]   bio[10]   bio[11]   bio[12]
## 19.130708 11.037544 12.874544  4.553622
```

Distance parameters .

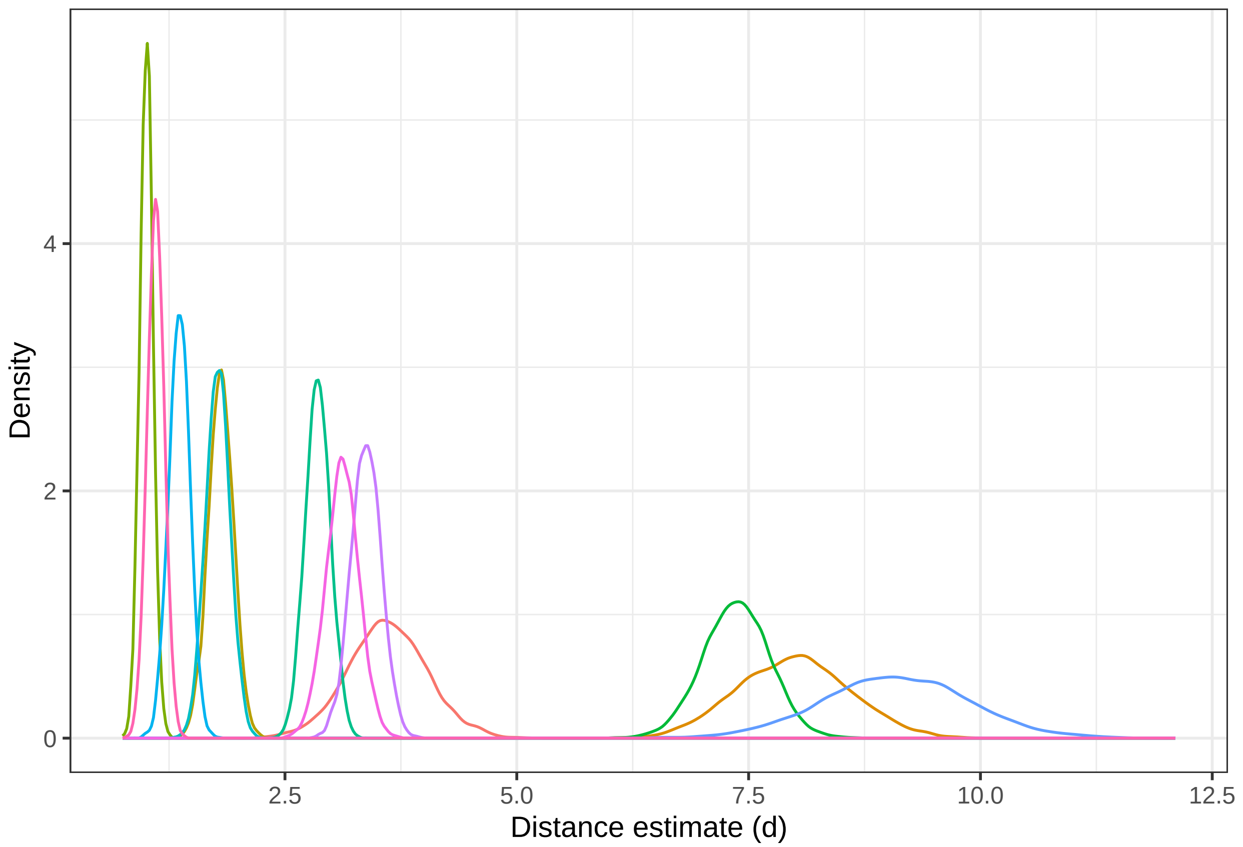

Supplementary Figure S10: posterior distribution of tree species distance effect on litterfall (each color represent a species).

Average estimate:

```
##     d[1]     d[2]     d[3]     d[4]     d[5]     d[6]     d[7]     d[8]
## 3.604296 7.366454 2.854762 1.776498 1.360425 9.083026 3.374464 3.118775
##     d[9]    d[10]    d[11]    d[12]
## 1.107232 7.986714 1.807896 1.006108
```

Biomass-distance interaction .

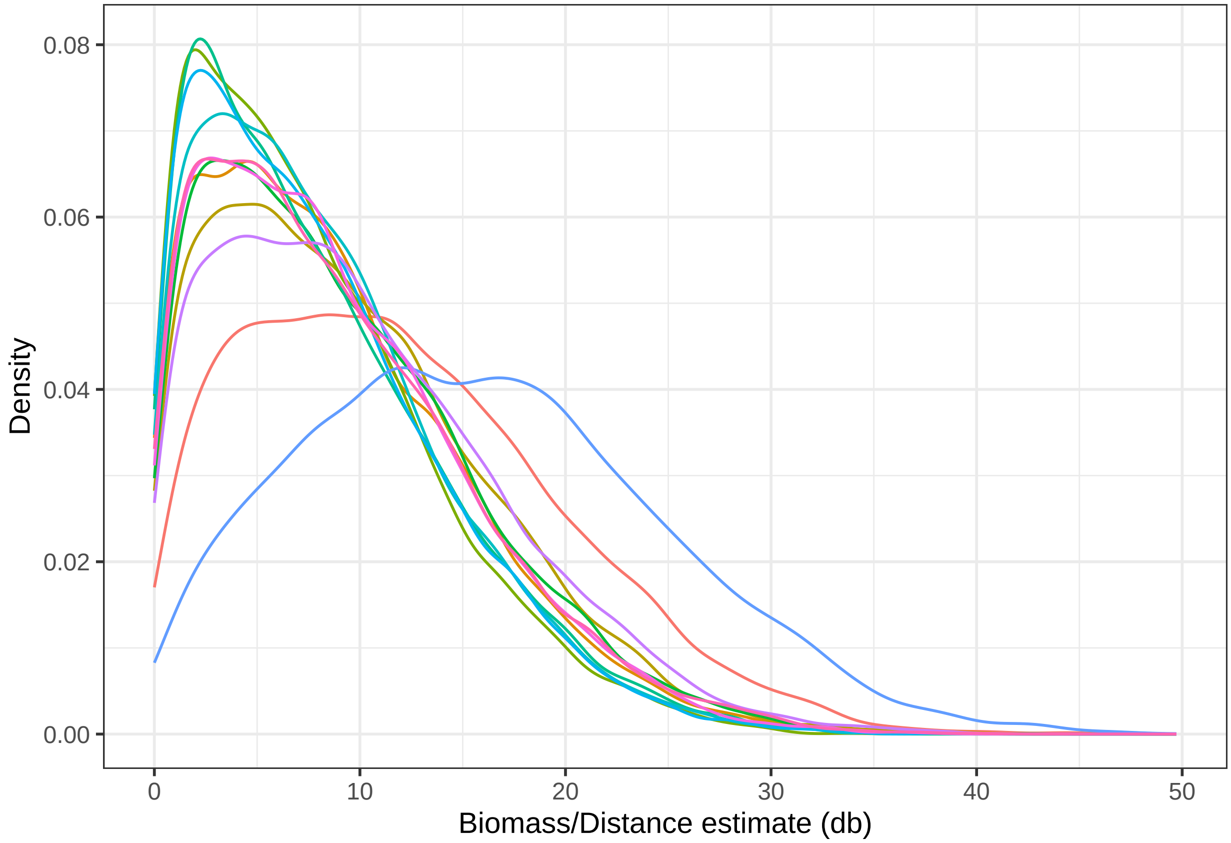

Supplementary Figure S11: posterior distribution of tree species biomass and distance interaction effect on litterfall (each color represent a species).

Average estimate:

```
##     db[1]     db[2]     db[3]     db[4]     db[5]     db[6]     db[7]     db[8]
## 11.998212  9.103719  8.085075  8.243736  7.992058 15.906301  9.995401  8.863504
##     db[9]    db[10]    db[11]    db[12]
##  8.932151  8.782380  9.558867  7.778018
```

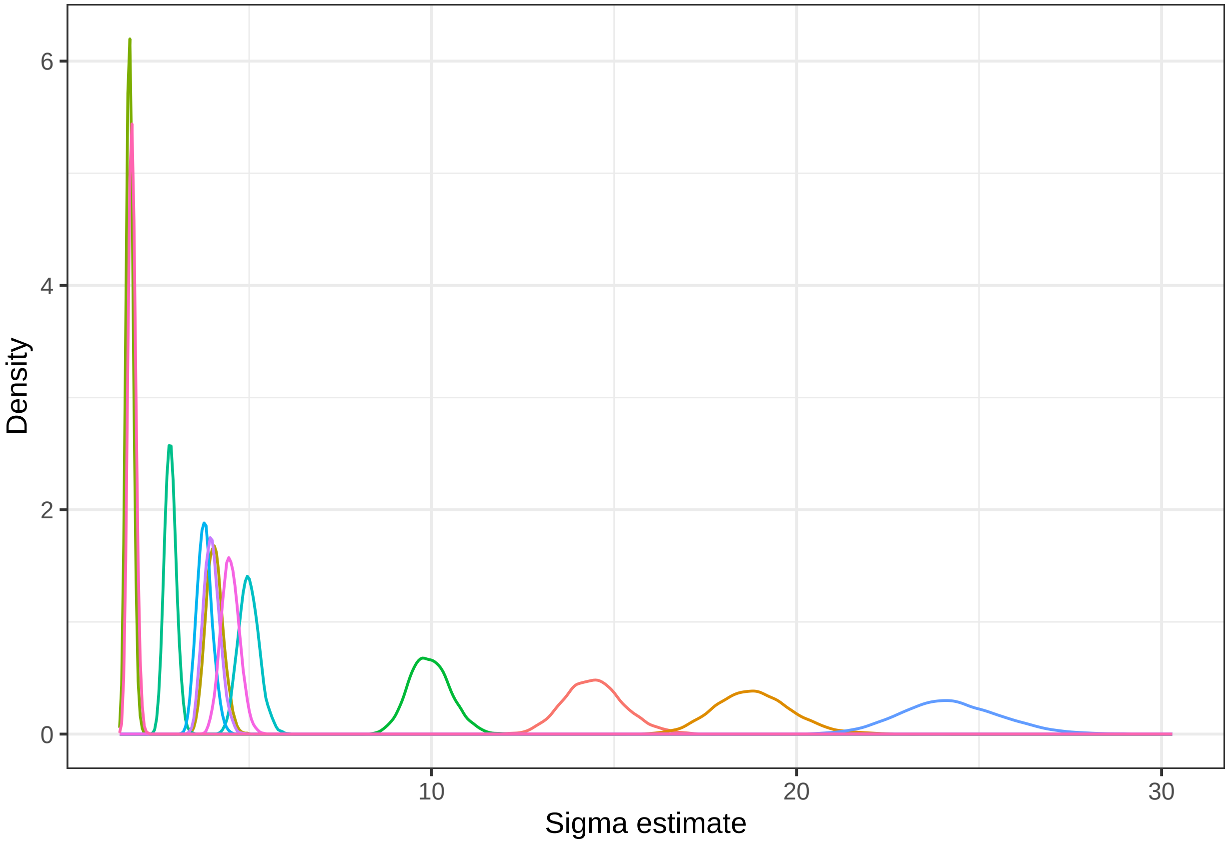

Sigma

Supplementary Figure S12: posterior distribution of sigma (each color represent a species).

Species-specific R2

```
##  [1] 0.3792272 0.7345575 0.7448733 0.6457222 0.5213376 0.5199647 0.7588883
##  [8] 0.7315600 0.5664792 0.5109918 0.5755203 0.5928872
```

```
## [1] 0.5630426
```

LOO value

```
##
## Computed from 8000 by 1920 log-likelihood matrix.
##
##          Estimate    SE
## elpd_loo  -6629.5 331.2
## p_loo       838.0 289.6
## looic     13259.0 662.3
## ------
## MCSE of elpd_loo is NA.
## MCSE and ESS estimates assume independent draws (r_eff=1).
##
## Pareto k diagnostic values:
##                          Count Pct.    Min. ESS
## (-Inf, 0.7]   (good)     1879  97.9%   151
##    (0.7, 1]   (bad)        14   0.7%   <NA>
##    (1, Inf)   (very bad)   27   1.4%   <NA>
```

```
## See help('pareto-k-diagnostic') for details.
```

Model predictions

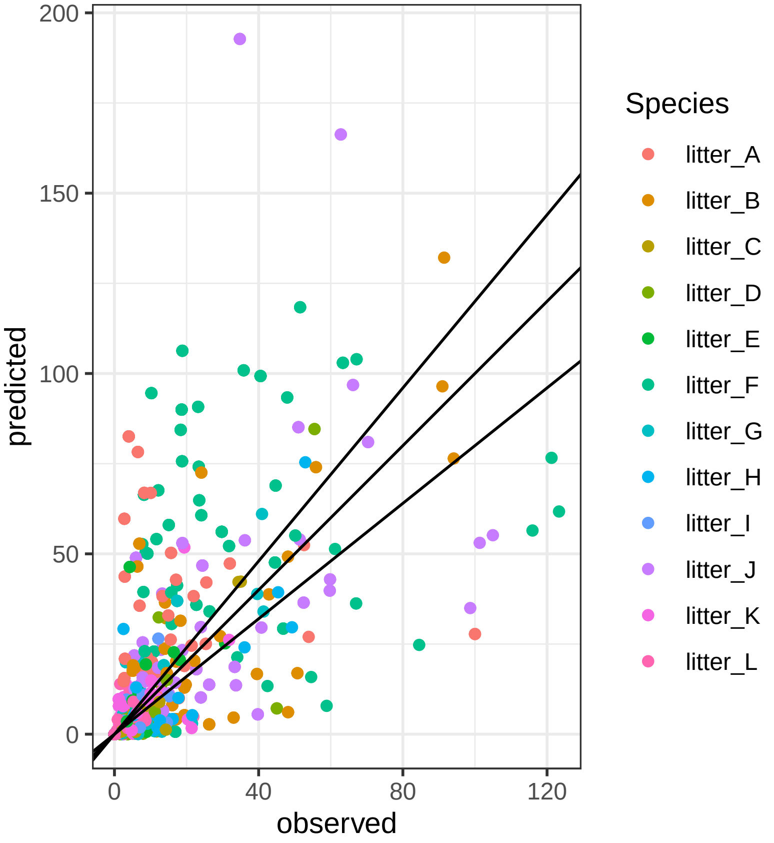

Supplementary Figure S13: Species specific litterfall posterior prediction (each color represents a species and the lines represent the 1:1 relationship and 95% interval).

Decomposition models

Input data

.

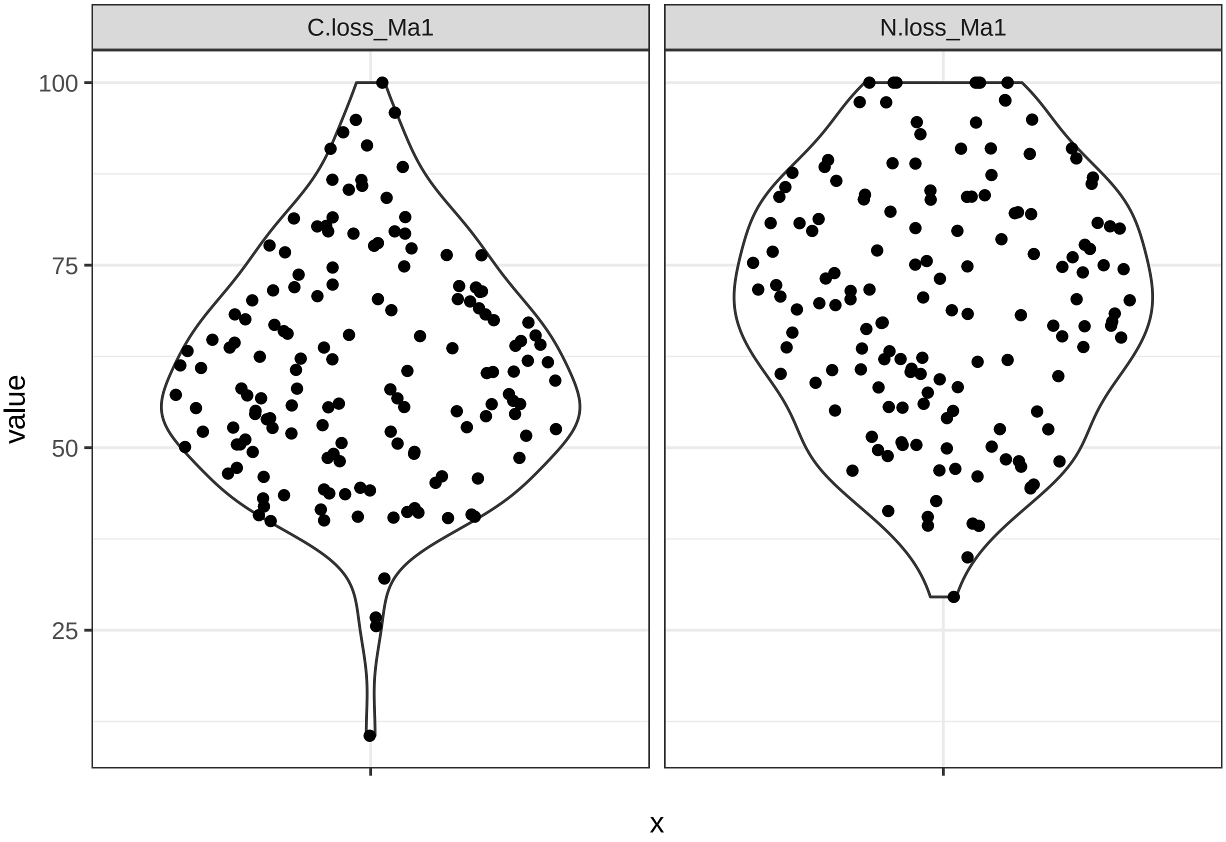

Supplementary Figure S14: Carbon and Nitrogen loss measured value distribution.

Species abundance

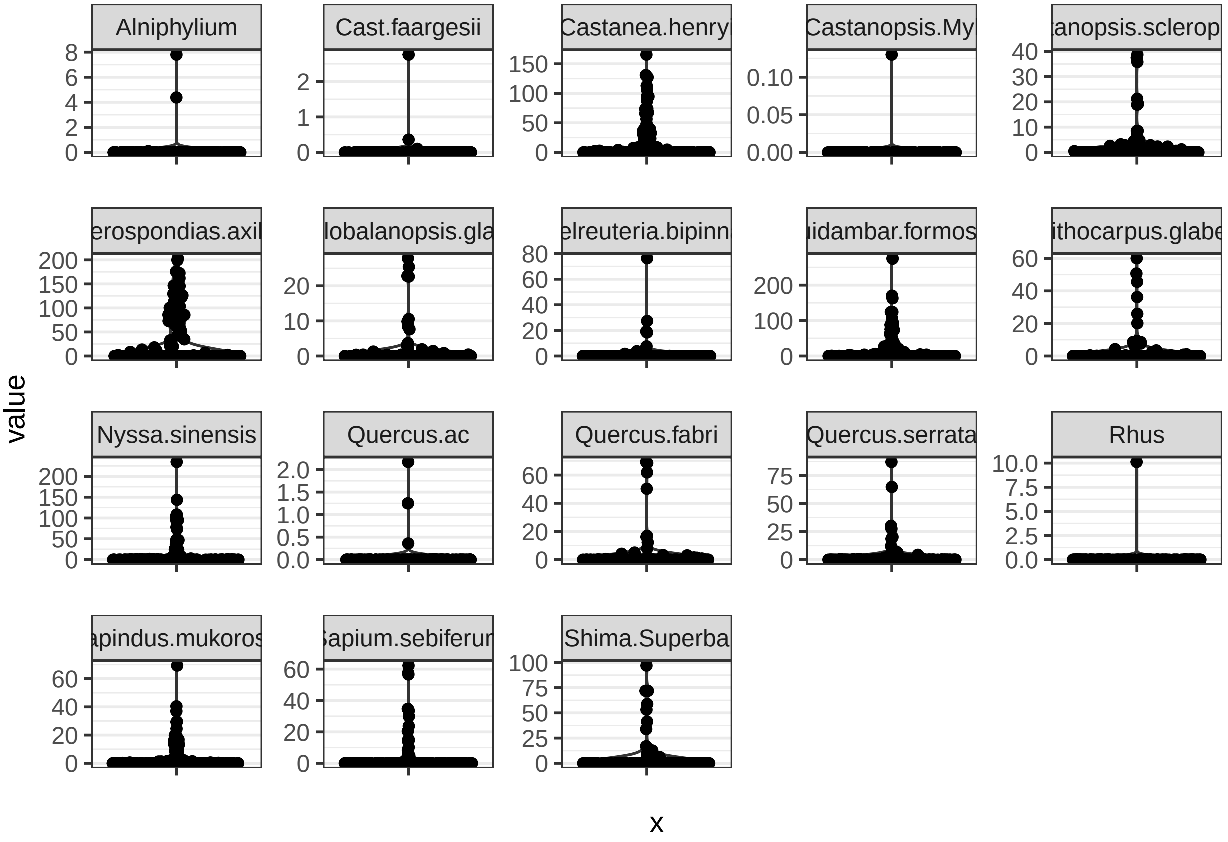

Supplementary Figure S15: Species-specific litter abundance.

Model structure

```
stan_code = '
data{
  int n; // observations
  int m; // species number
  vector[n] decompC; // carbon decomposition
  vector[n] litterBM; // total litter biomass
  vector[n] litterSR; // litter species richness
  matrix[n, m] p; // proportion of species in litter biomass
}

parameters{
  vector[m] b; // species specific proportion parameter
  vector[m] a; // species specific interraction parameter
  real c; // litter biomass parameter
  real d; // litter species richness parameter
  real<lower=0> sigma; // sigma
}

model{
  // auxiliary variables
  real speff;
  real intereff;
```

```
  // Priors
  b ~ normal(0,10); // species specific proportion parameter
  a ~ normal(0,10); // species specific interraction parameter
  c ~ normal(0,10); // litter biomass parameter
  d ~ normal(0,10); // litter species richness parameter
  sigma ~ normal(0,10); // sigma

  // Likelihood

  for(k in 1:n){
    speff = 0.0;
    intereff = 0.0;
    for(i in 1:m){
      // summed species effects
      speff = speff + b[i] * p[k,i];
      // summed interaction effects: use trick for avoiding double sums
      intereff = intereff + a[i]*p[k,i]*(1-p[k,i]);
     }
    decompC[k] ~ normal(speff + intereff + c * litterBM[k] + d * litterSR[k], sigma);
  }
}
'
```

Model fit

Model R2 Carbon

```
## [1] 0.4906167
```

Nitrogen

```
## [1] 0.5363231
```

Posterior distribution

Carbon model .

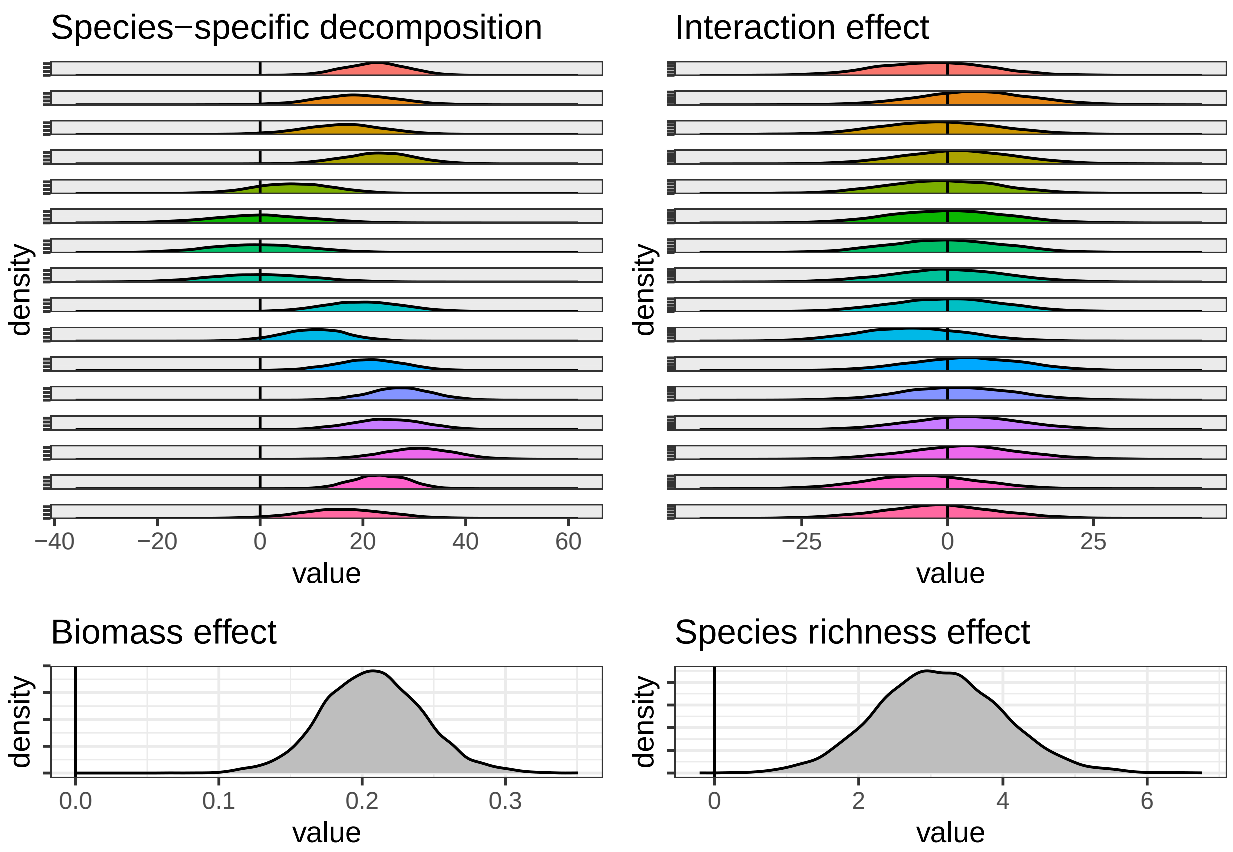

Supplementary Figure S16: Parameters posterior distribution for the carbon loss model.

Nitrogen model .

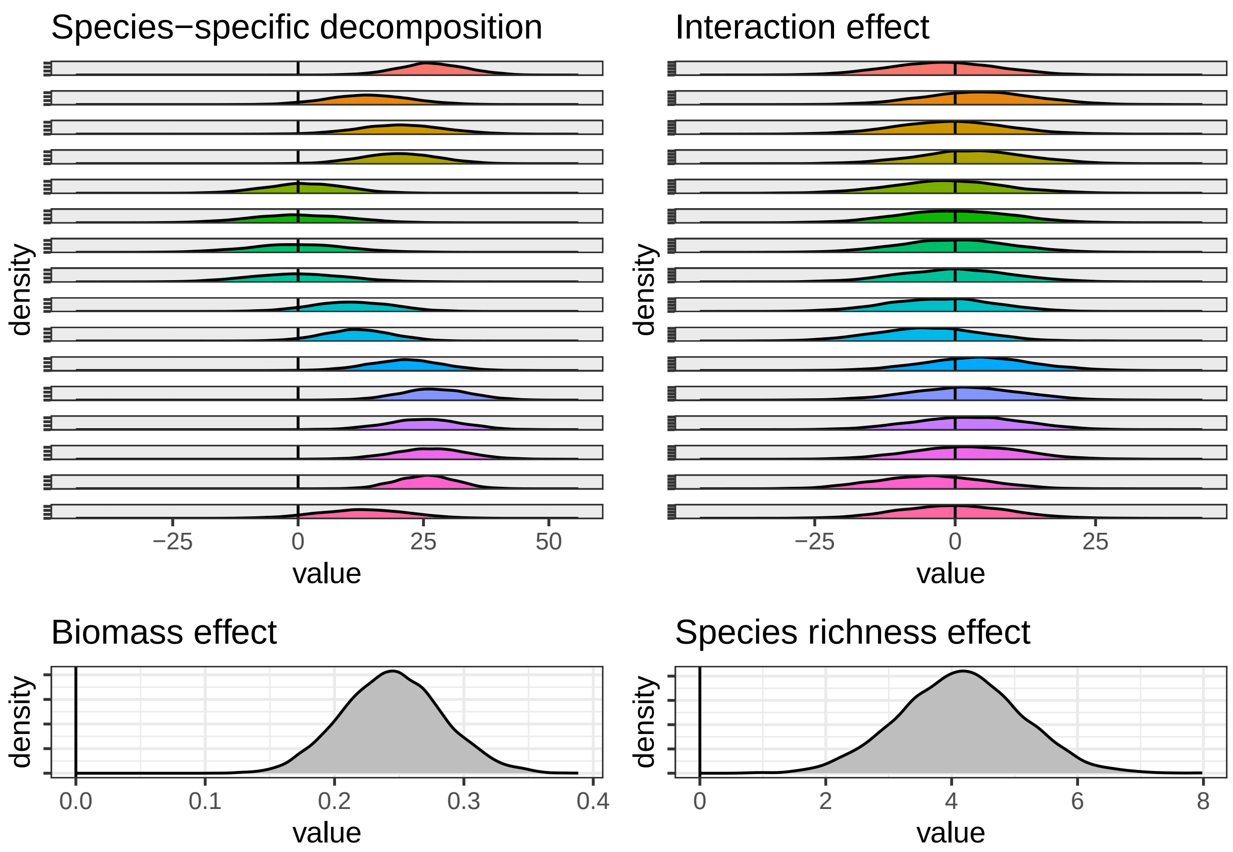

Supplementary Figure S17: Parameters posterior distribution for the nitrogen loss model.

Model predictions

Carbon

.

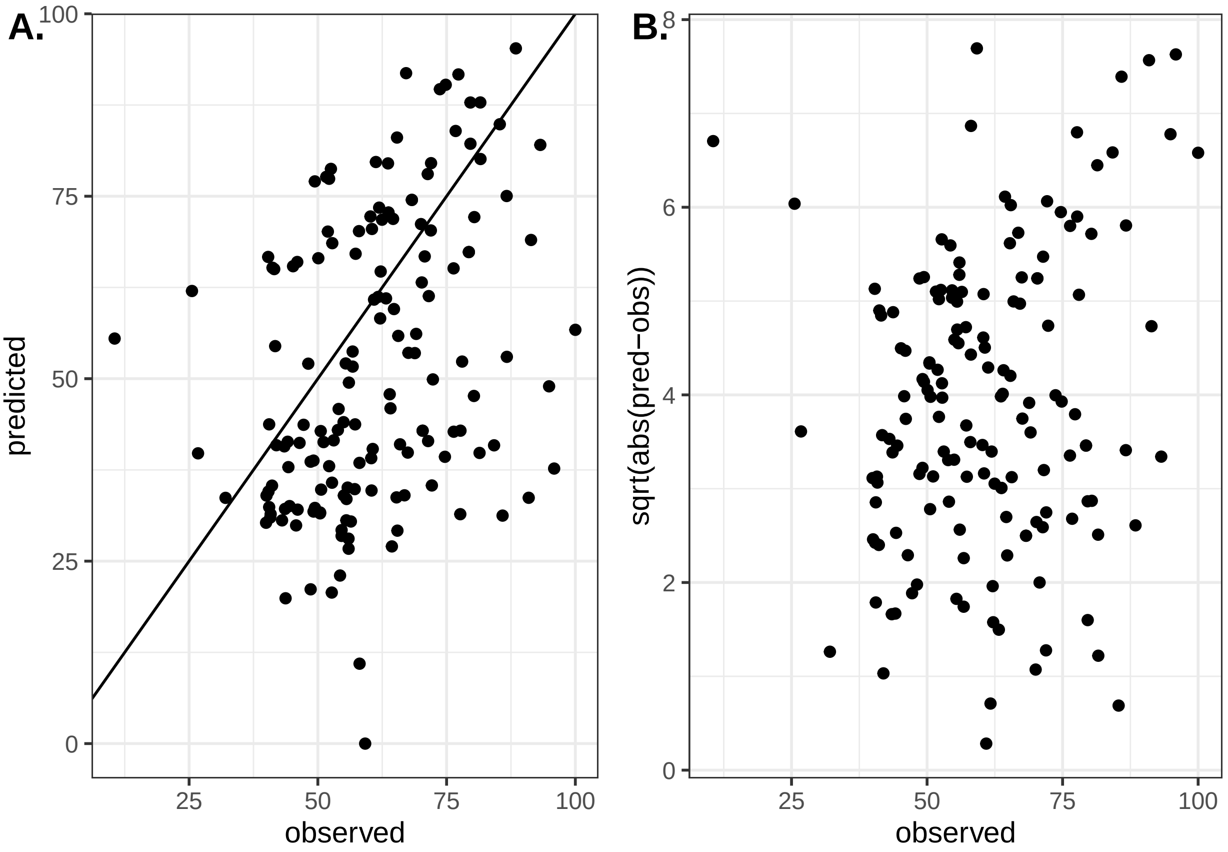

Supplementary Figure S18: carbon loss model predictions. A. Predicted vs. Observed and B. Root squared error distribution.

Nitrogen

.

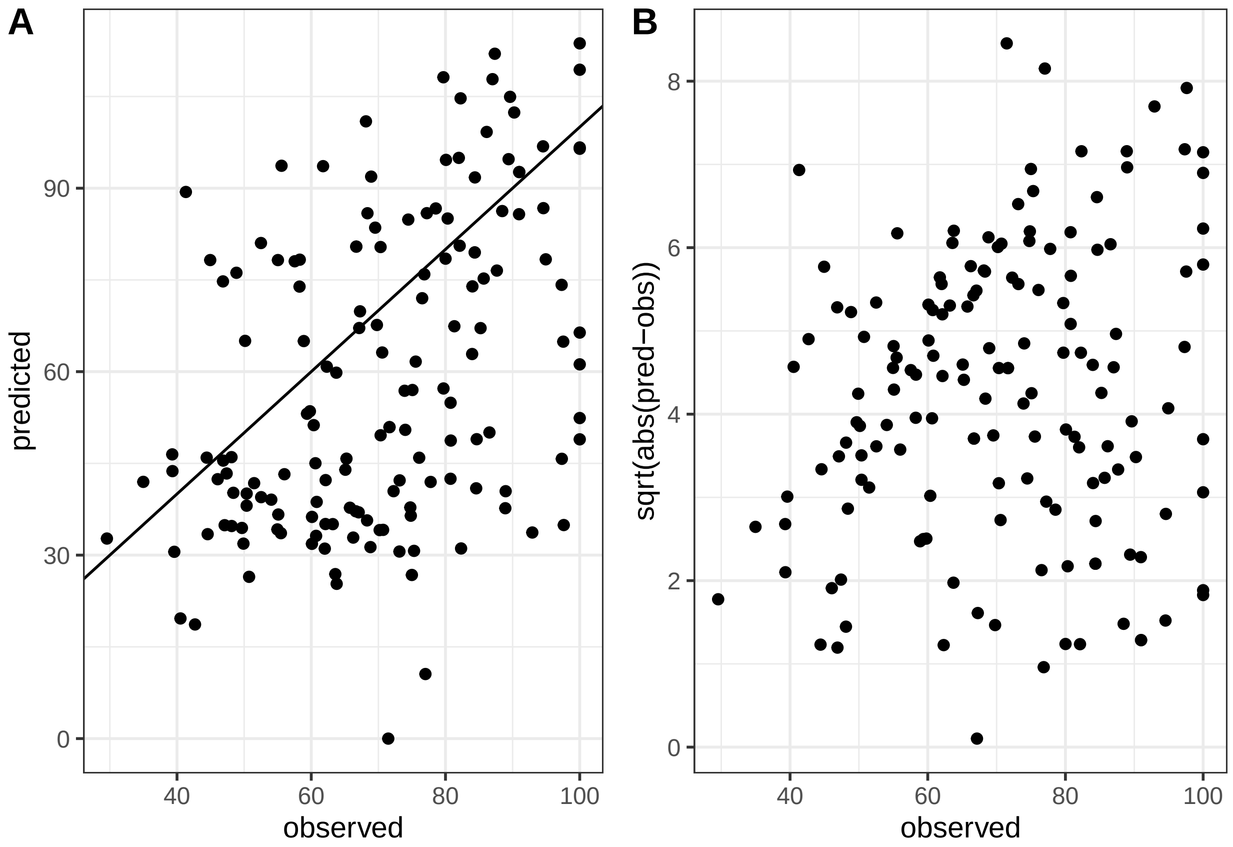

Supplementary Figure S19: nitrogen loss model predictions. A. Predicted vs. Observed and B. Root squared error distribution.

Supplementary Note S3: nitrogen decomposition results

Simulation outputs

Eight species mixtures additional variabes

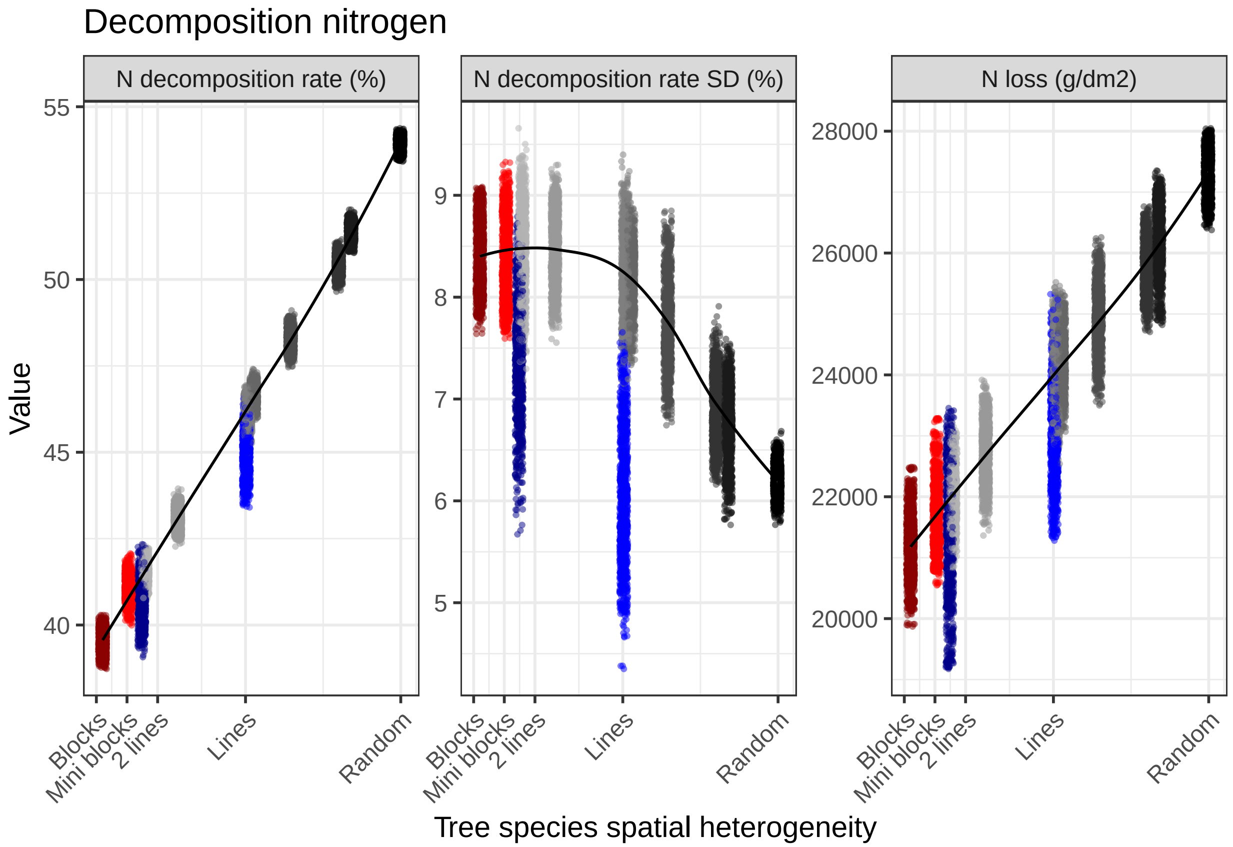

Supplementary Figure S20: Interaction between tree species richness and tree species spatial heterogeneity on nitrogen decomposition from two species mixture (n = 56) to four (n = 1,000) and eight-species mixtures (n = 1,000) using an 8 levels gradient of heterogeneity ranging from a block (in red) to a fully random design (grey color gradient). The loess regression lines were added for each heterogeneity level, and grey ribbons highlight the 95% confidence interval around the mean values. Model ANOVA outputs are reported using: “sp” = tree species richness, “h” = tree species spatial heterogeneity, and “sp x h” interaction between the two variables.
