# Supplementary material for: Improving forest ecosystem functions by optimizing tree species spatial arrangement
Source: Nat Commun. 2025 Jul 9;16:6286. doi: 10.1038/s41467-025-61389-7 (PMC12241584; doi:10.1038/s41467-025-61389-7)
Supplement: Supplementary file 2 — Reporting Summary [file 41467_2025_61389_MOESM2_ESM.pdf]

nature portfolio

Corresponding author(s): Rémy Beugnon

Last updated by author(s): May 9, 2025

Reporting Summary

Nature Portfolio wishes to improve the reproducibility of the work that we publish. This form provides structure for consistency and transparency in reporting. For further information on Nature Portfolio policies, see our Editorial Policies and the Editorial Policy Checklist.

Statistics

For all statistical analyses, confirm that the following items are present in the figure legend, table legend, main text, or Methods section.

n/a	Confirmed	
☐	☒	The exact sample size (*n*) for each experimental group/condition, given as a discrete number and unit of measurement
☐	☒	A statement on whether measurements were taken from distinct samples or whether the same sample was measured repeatedly
☐	☒	The statistical test(s) used AND whether they are one- or two-sided<br>*Only common tests should be described solely by name; describe more complex techniques in the Methods section.*
☐	☒	A description of all covariates tested
☐	☒	A description of any assumptions or corrections, such as tests of normality and adjustment for multiple comparisons
☐	☒	A full description of the statistical parameters including central tendency (e.g. means) or other basic estimates (e.g. regression coefficient) AND variation (e.g. standard deviation) or associated estimates of uncertainty (e.g. confidence intervals)
☐	☒	For null hypothesis testing, the test statistic (e.g. *F*, *t*, *r*) with confidence intervals, effect sizes, degrees of freedom and *P* value noted<br>*Give P values as exact values whenever suitable.*
☒	☐	For Bayesian analysis, information on the choice of priors and Markov chain Monte Carlo settings
☒	☐	For hierarchical and complex designs, identification of the appropriate level for tests and full reporting of outcomes
☒	☐	Estimates of effect sizes (e.g. Cohen's *d*, Pearson's *r*), indicating how they were calculated

Our web collection on statistics for biologists contains articles on many of the points above.

Software and code

Policy information about availability of computer code

Data collection	The data used and generated in this study have been deposited in the Zenodo database under accession code 10.5281/zenodo.13808826
Data analysis	The RScripts used in this study have been deposited in the Zenodo database under accession code 10.5281/zenodo.13808826

For manuscripts utilizing custom algorithms or software that are central to the research but not yet described in published literature, software must be made available to editors and reviewers. We strongly encourage code deposition in a community repository (e.g. GitHub). See the Nature Portfolio guidelines for submitting code & software for further information.

Data

Policy information about availability of data

All manuscripts must include a data availability statement. This statement should provide the following information, where applicable:

- Accession codes, unique identifiers, or web links for publicly available datasets
- A description of any restrictions on data availability
- For clinical datasets or third party data, please ensure that the statement adheres to our policy

Data and simulation codes are provided for readers using Zenodo stable repository: 10.5281/zenodo.13808826

Research involving human participants, their data, or biological material

Policy information about studies with human participants or human data. See also policy information about sex, gender (identity/presentation), and sexual orientation and race, ethnicity and racism.

Reporting on sex and gender	Not relevant for the study
Reporting on race, ethnicity, or other socially relevant groupings	Not relevant for the study
Population characteristics	Not relevant for the study
Recruitment	Not relevant for the study
Ethics oversight	Not relevant for the study

Note that full information on the approval of the study protocol must also be provided in the manuscript.

Field-specific reporting

Please select the one below that is the best fit for your research. If you are not sure, read the appropriate sections before making your selection.

☐ Life sciences ☐ Behavioural & social sciences ☒ Ecological, evolutionary & environmental sciences

For a reference copy of the document with all sections, see nature.com/documents/nr-reporting-summary-flat.pdf

Ecological, evolutionary & environmental sciences study design

All studies must disclose on these points even when the disclosure is negative.

Study description	To study the effect of tree spatial heterogeneity on forest productivity and litter dynamics (litterfall and litter decomposition), we simulated these processes across a range of plantation designs using inventories, litterfall collections, and leaf litter decomposition field data. In short, we simulated two-, four-, and eight-species mixture forests from a pool of 8 tree species of a Biodiversity-Ecosystem Functioning experiment in subtropical China (BEF-China). For all the possible mixture combinations, we selected all 28 two-species mixture permutations, 1,000 four-, and 1,000 eight-species mixtures permutations. Each forest from these 2,132 species mixture permutations was “planted” (i.e. simulated) in forest stands of 16 individuals by 16 individuals of trees planted at a one-meter distance (17 x 17 m forests) with eight different types of spatial distributions from blocks of species to fully random distributions of the species. In addition, eight-species mixtures were also planted in smaller blocks, double rows, and single rows of species, to better represent realistic plantation design. From tree and species-specific Bayesian models fitted on our experimental litterfall and decomposition data, we simulated the litterfall distribution of each tree and litter decomposition for each 10 x 10 cm pixel in the simulated forest.
Research sample	The complete simulation pipeline is available for readers on the Zenedo repository. Species biomass production models were extracted from Wang et al (2023) and species-specific litterfall and decomposition data were extracted from Beugnon et al. (2023) and added into the Zenodo folder.
Sampling strategy	Sampling strategy of litterfall and decomposition were defined by Beugnon et al. (2023), simulation sampling strategy was to replicate each potential tree community at least 2 times (all 28wo-species mixture permutations, 1,000 four-, and 1,000 nine-species mixtures permutations).
Data collection	Data were collected from Beugnon et al 2023 and thereafter simulated.<br><br>Tree biomass estimations<br>Tree biomass, which was used for fitting litterfall models, was predicted for all tree pairs and their neighbors using tree basal area (BA) and species-specific allometric relationships estimated on the pair of trees. (1) Circumference at breast height (CBH) was measured in September 2018 for all pairs of trees and their direct neighbors in order to calculate the basal area of these trees as = ( )2/4 . (2) Tree height was measured for the pair of trees, and tree biomass was calculated following Huang et al. (2017). Tree pairs’ BA and biomass were used to estimate species-specific allometric BA-biomass relationships and predict the tree biomass for all neighboring trees (Beugnon, Bu, et al., 2023).<br>Litterfall sampling<br>In September 2018, a litter trap of 1 m^2 was set up at a height of 1 m above the soil surface between each pair of trees (see Beugnon, Eisenhauer, et al., 2023). Litter was collected during December 2018 to cover the main litterfall season in the region (Huang et al., 2017). To measure litterfall composition, each leaf of the litter trap was sorted and identified to species level. Each species' litter was dried at 40°C for two days and weighed (± 0.1 g).<br>Decomposition measurements<br>We performed a decomposition experiment between the pair of trees to measure<br>total leaf litter decomposition. Large-mesh litter bags (10 cm x 10 cm) were built using a 5 mm-mesh for the upper part of the bag to provide access to macro-decomposers, and a 0.054 mm-mesh at the bottom to prevent loss of fine leaf litter particles, and filled with 2 g (± 0.01 g) of dried litter according to litter trap species composition (i.e., species-specific biomasses) of the different pairs of trees. Therefore, the litter composition of the litterbags matched exactly the litter composition (i.e., species-specific litter masses) collected

in the litter traps of the corresponding pair of trees. The litterbags were installed in December 2018 and covered by a 1 m x 1 m grid to prevent dislocation by heavy rainfalls (1 cm mesh size). In September 2019, i.e., after nine months of decomposition and before the start of litterfall, litterbags were collected, water-cleaned and dried at 40°C for two days. The residual litter was weighed (± 0.01 g) and milled.
Litter C and N content after decomposition were measured from the residual litter with an elemental analyzer (Vario EL Cube, Elementar, Langenselbold, Germany) and corrected for soil contamination following Beugnon, Eisenhauer et al. (2023). C and N loss rates (%) from the litterbags during the period from December 2018 to September 2019 were calculated.

Timing and spatial scale	Data from Beugnon et al 2023 were collected from September to December 2018 for litterfall (i.e. main litterfall period) and decomposition was measured from December 2018 to September 2019 (9 months)
Data exclusions	Only species available in both Wang et al 2024 and Beugnon et al 2023 were selected for this study.
Reproducibility	All data used and simulation pipeline are available in the Zenodo folder
Randomization	Full range of potential communities compositions were simulated at least twice.
Blinding	Communities were randomly selected for simulations

Did the study involve field work? ☒ Yes ☐ No

Field work, collection and transport

Field conditions	The region is characterized by a subtropical climate with warm, rainy summers and cool, dry winters with a mean temperature of 16.7°C and a mean annual rainfall of 1821 mm (Yang et al., 2013). Soils in the region are Cambisols and Cambisol derivatives, with Regosol on ridges and crests (Geißler et al., 2012; Scholten et al., 2017). The natural vegetation consists of species-rich broad-leaved forests dominated by Cyclobalanopsis glauca, Castanopsis eyrei, Daphniphyllum oldhamii, and Lithocarpus glaber (Bruelheide et al., 2011, 2014).
Location	The study site is located in southeast China near Xingangshan city (Jiangxi Province, 29.08–29.11° N, 117.90–117.93° E). Our experimental site is part of the BEF-China experiment (site A, Bruelheide et al., 2014), and it was planted in 2009 after a clearcut of the previous commercial plantation.
Access & import/export	The sampling was part of the sino-german research program TreeDi
Disturbance	na

Reporting for specific materials, systems and methods

We require information from authors about some types of materials, experimental systems and methods used in many studies. Here, indicate whether each material, system or method listed is relevant to your study. If you are not sure if a list item applies to your research, read the appropriate section before selecting a response.

Materials & experimental systems

n/a	Involved in the study	
☒	☐	Antibodies
☒	☐	Eukaryotic cell lines
☒	☐	Palaeontology and archaeology
☒	☐	Animals and other organisms
☒	☐	Clinical data
☒	☐	Dual use research of concern
☐	☒	Plants

Methods

n/a	Involved in the study	
☒	☐	ChIP-seq
☒	☐	Flow cytometry
☒	☐	MRI-based neuroimaging

Dual use research of concern

Policy information about dual use research of concern

Hazards

Could the accidental, deliberate or reckless misuse of agents or technologies generated in the work, or the application of information presented in the manuscript, pose a threat to:

No	Yes	
☒	☐	Public health
☒	☐	National security
☒	☐	Crops and/or livestock
☒	☐	Ecosystems
☒	☐	Any other significant area

Experiments of concern

Does the work involve any of these experiments of concern:

No	Yes	
☒	☐	Demonstrate how to render a vaccine ineffective
☒	☐	Confer resistance to therapeutically useful antibiotics or antiviral agents
☒	☐	Enhance the virulence of a pathogen or render a nonpathogen virulent
☒	☐	Increase transmissibility of a pathogen
☒	☐	Alter the host range of a pathogen
☒	☐	Enable evasion of diagnostic/detection modalities
☒	☐	Enable the weaponization of a biological agent or toxin
☒	☐	Any other potentially harmful combination of experiments and agents

Plants

Seed stocks	Not relevant
Novel plant genotypes	Not relevant
Authentication	Not relevant
